# Anti-Inflammatory and Anticancer Activities of Taiwanese Purple-Fleshed Sweet Potatoes (*Ipomoea batatas* L. Lam) Extracts

**DOI:** 10.1155/2015/768093

**Published:** 2015-10-05

**Authors:** Marcelia Sugata, Chien-Yih Lin, Yang-Chia Shih

**Affiliations:** Department of Biotechnology, Asia University, 500 Liufeng Road, Wufeng District, Taichung 41354, Taiwan

## Abstract

Purple-fleshed sweet potato (PFSP) (*Ipomoea batatas* L. Lam) has been known to possess high amount of anthocyanins which contribute to its antioxidant activity. However, a few reports are available concerning its anti-inflammatory and anticancer properties. In this study, PFSP “Tainung 73,” which is locally grown in Taiwan, was steamed and extracted using acidified ethanol pH 3.5 under 80°C. Two kinds of crude anthocyanins extracts were obtained, namely, SP (Steamed, Peeled) and SNP (Steamed, No Peeled). Then, anti-inflammatory and anticancer activities of these extracts were investigated. Cell viability assay (MTT) showed that SP and SNP extracts were not toxic to RAW 264.7 cells. They even exhibited anti-inflammatory activities by suppressing the production of NO and proinflammatory cytokines, such as NF-*κβ*, TNF-*α*, and IL-6, in LPS-induced macrophage cells. Anticancer activities of these extracts were displayed through their ability to inhibit the growth of cancer cell lines, such as MCF-7 (breast cancer), SNU-1 (gastric cancer), and WiDr (colon adenocarcinoma), in concentration- and time-dependent manner. Further studies also revealed that SP extracts could induce apoptosis in MCF-7 and SNU-1 cancer cells through extrinsic and intrinsic pathway. In the future, PSFP extracts may have potential to be applied in nutraceutical, pharmaceutical, and food industries.

## 1. Introduction

Purple sweet potato (*Ipomoea batatas* (L.) Lam) is a dicotyledonous plant that belongs to the family Convolvulaceae [1]. It ranks as the seventh important staple crop in the world and the fifth in developing countries after rice, wheat, maize, and cassava [2]. Because it has enormous genetic diversity of both phenotypic and morphological traits [3], the crop has great potential for further development to accommodate specific uses [4]. Tainung 73 (TNG 73) is one of the newly cultivated purple sweet potatoes developed by breeders of Taiwan Agricultural Research Institute (TARI).

Over the past years, phytochemicals in plants have recently attracted great attention from research communities, food industries, and consumers. Many scientific papers have reported that phytochemicals, such as phenolics, flavonoids, and anthocyanins, in fruit and vegetable possess high antioxidant activities that can reduce oxidative damage caused by reactive oxygen species (ROS) [5, 6]. Hence, phytochemicals play important role in preventing chronic diseases that are related to oxidative stress caused by free radicals, such as cancer, inflammation, atherosclerosis, and ageing [7–9].

Purple sweet potatoes have been known to possess high amount of anthocyanins in the storage root, of which cyanidin and peonidin are major anthocyanidins [10–13]. Root tuber of TNG 73 is also rich in anthocyanins and the concentration is higher in root skin than in root flesh [14]. Previous studies have reported that anthocyanins demonstrated ability to protect against a myriad of human diseases such as liver dysfunction, hypertension, vision disorders, microbial infections, and diarrhea [15–17]. Due to this fact, a high intake of anthocyanin-rich food has been associated with health preventive effects and reduced risks of age-related macular degeneration [18], anticancerogenic activity [19], antioxidant capacity [20–23], antiulcer activity [24], and also reduced risks of cardiovascular disorders [25].

Human body constantly reacts with oxygen as it breathes and its cells produce energy. As a consequence of this activity, highly reactive molecules are produced within our cells known as free radicals and oxidative stress occurs. Furthermore, excessive oxidative stress may lead to inflammation and even cancer [26]. In this case, antioxidative compounds which can suppress oxidative stress might also have anti-inflammatory and anticancer activity. So far, among all health benefits of phytochemicals in purple sweet potatoes, their free radical scavenging and antioxidant capacities are the most widely publicized. However, their anti-inflammatory and anticancer activities have not been well studied.

## 2. Experimental Section

### 2.1. Preparation of PFSP TNG 73 Extracts

Root tuber of TNG 73 was steamed for 60 minutes at 90°C and then cleaned, peeled (Steamed, Peeled (SP)) or not peeled (Steamed, No Peeled (SNP)), and cut into 2-3 cm thick slices followed by smashing until being even delicate. Smashed samples were added into sterile 50 mL conical Falcon tubes and then extracted with acidified ethanol pH 3.5. The ratio of PFSP weight per solvent volume was 17% (w/v), in which each 6.8-gram fresh weight PFSP was extracted with 40 mL solvent [27]. All tubes were incubated for 1 hour in water bath with 1,000 rpm shaking at temperature of 80°C. The supernatant was removed using 0.45 *μ*m syringe filter and the solvent was evaporated using rotary evaporator. The extracts were subsequently stored overnight at −80°C and freeze-dried for 3–5 days. The dried extracts were kept at −20°C before being further used.

### 2.2. MTT Assay

The effect of PFSP extracts on the viability of macrophage and cancer cells was determined using MTT assay. This is a colorimetric assay that measures the reduction of yellow 3-(4,5-dimethylthiazol-2-yl)-2,5-diphenyltetrazolium bromide (MTT) by mitochondrial succinate dehydrogenase. MTT enters the cells and passes into the mitochondria where it is reduced to an insoluble, colored (dark purple) formazan product [28]. Formazan can be solubilized with an organic solvent and then measured spectrophotometrically. Since MTT reduction only occurs in metabolically active cells, their activity is a measure of cell viability.

Based on the method described previously, 100 *μ*L of RAW 264.7 (5 × 10^4^, 1 × 10^5^, and 2 × 10^5^), MCF-7 (5 × 10^4^), WiDr (5 × 10^4^), and SNU-1 (5 × 10^4^) cells/mL was cultured in 96-well microculture plate (Nunc, Denmark) for 24 h. The cells were pretreated by PFSP extracts (0.1, 0.5, 1, 2, and 3 mg/mL for RAW 264.7 and 1, 2, 3, 4, 5, and 6 mg/mL for cancer cells) for 24 h, 48 h, and 72 h subsequently after the cells were washed twice with phosphate buffer saline (PBS). Then, 10 *μ*L of MTT dye (5 mg/mL) was added to the wells. After 4 h, 100 *μ*L of SDS-HCl was added to all the wells to dissolve the formazan crystals and the absorbance was measured at 590 nm. Cell viability was calculated using the following:

(1) $$\begin{array}{c} \text{Cell viability}\left(\;\%\;\right)=\left(\;\frac{{\text{A}}_{\text{sample}}}{{\text{A}}_{\text{control}}}\;\right)\times 100\%, \end{array}$$

where A_sample_ and A_control_ are the absorbance from the mixture with and without the addition of test sample, respectively. The assays were carried out in triplicate and expressed as mean values ± Standard Deviation (SD).

### 2.3. Migration Assay

The study of cell migration* in vitro* was done by using Migration Assay [29], which observes the cell migration into a “wound” that is created on a cell monolayer. RAW 264.7 and MCF-7 cells were grown in 12-well plates until 90% confluent (2 × 10^5^ cells/mL) and then scratched to form a “wound” using sterile pipette tips. The cells were then cultured in the presence or absence of LPS (1 *μ*g/mL) and/or PFSP extracts (1, 2, and 3 mg/mL for RAW 264.7 and 1, 3, and 5 mg/mL for MCF-7) for 48 h. The images were recorded at 0, 24, and 48 h after the scratch using a light microscope.

### 2.4. Nitric Oxide (NO) Griess Assay

RAW 264.7 cells were placed in a 12-well plate at a density of 5 × 10^5^ cells/mL and incubated for 24 h. Cultured cells were treated with various concentrations of PFSP extracts (1, 2, 3, 4, and 5 mg/mL) with 1 *μ*g/mL bacterial lipopolysaccharides (LPS,* E. coli* 0111:B4, Difco, Detroit, MI, USA) for 24 h. The level of nitric oxide (NO) production in cell culture supernatants was determined using a colorimetric assay based on Griess reaction [30]. Aliquots of 100 *μ*L of supernatants were mixed with 100 *μ*L Griess reagent (50 *μ*L of 1% sulfanilamide in 5% phosphoric acid and 50 *μ*L of 0.1% naphthyl-ethylenediamine dihydrochloride). After 10 min, the absorbance was determined at 540 nm.

### 2.5. Western Blot

Cells (RAW 264.7, MCF-7, and SNU-1) were seeded at a density of 2 × 10^5^ cells/mL in a 10 mL dish and incubated for 24 h. Cells were then treated with LPS and/or PFSP extracts for certain time (18 h for RAW 264.7 and 24 h for MCF-7 and SNU-1). Proteins were extracted using RIPA (Radioimmunoprecipitation Assay) buffer containing 1% proteinase inhibitor (Thermo Scientific) and then quantified using Bradford reagent (Protein Assay Dye Reagent Concentrate, BIO-RAD) with bovine serum albumin as a standard. To determine the protein expression in the cytoplasm and the nuclei, western blot of cell lysate was performed according to the minor modification of the procedure described previously [31]. After boiling the sample for 5 min at 100°C, 30 *μ*g of protein was loaded per lane on acrylamide gel (12.5%) and subjected to sodium dodecyl sulfate-polyacrylamide gel electrophoresis (SDS-PAGE) at 80 V for 30 min and then at 110 V for 60 min. The ladder used in this study was BlueRay Prestained Protein Ladder from Gene Direx. Proteins in the gel were transferred by wet blotting onto Polyvinylidene Fluoride Transfer Membranes 0.45 *μ*m (PVDF) (PALL Corporation); then, the membranes were incubated overnight at 4°C or 60 min at RT in blocking buffer (5% nonfat dry milk in TBST). After washing, membranes were incubated overnight with primary antibody (1 : 2,000) in TBST at 4°C with gentle shaking and then with the secondary antibody (1 : 10,000) in TBST for 1 h at RT. All antibodies used in this study were purchased from Santa-Cruz Biotech, Inc. Before bands were detected using Western Bright ECL HRP substrate (Advansta), the membranes were washed in TBST five times for 5 min.

### 2.6. Statistical Analysis

Comparisons among treatment groups were made with the paired *t*-test (two groups) or the repeated measures of analysis of variance (more than two groups) using Duncan's test SPSS Statistics 21 version. All *P* values are two-tailed and the significance levels are ^*∗*^ *P* < 0.05, ^*∗∗*^ *P* < 0.01, and ^*∗∗∗*^ *P* < 0.001.

## 3. Results and Discussion

### 3.1. Effect of PFSP Extracts on RAW 264.7 Cell Viability

PFSP has been known to possess the highest anthocyanin contents among all flesh colors [32]. The most abundant anthocyanidin in Taiwanese PFSP TNG 73 has not been well identified, but it is often mentioned that cyanidin-3-glucoside was counted as the major anthocyanin in PFSP. Besides, Lewis et al. [33] reported that PFSP tubers contained mostly phenolic acids and small amount of flavonoids. Many studies showed polyphenol, flavonoid, and its derivatives (including anthocyanin) had a significant promotive effect on cell proliferation [34, 35]. However, some reports indicated that anthocyanin also showed an inhibitory effect on proliferation of cells [36–40]. The different results of anthocyanin on cell proliferation might be due to different targeted cells and their number and different dosages, sources, and preparation methods of anthocyanin.

**Figure 1:**
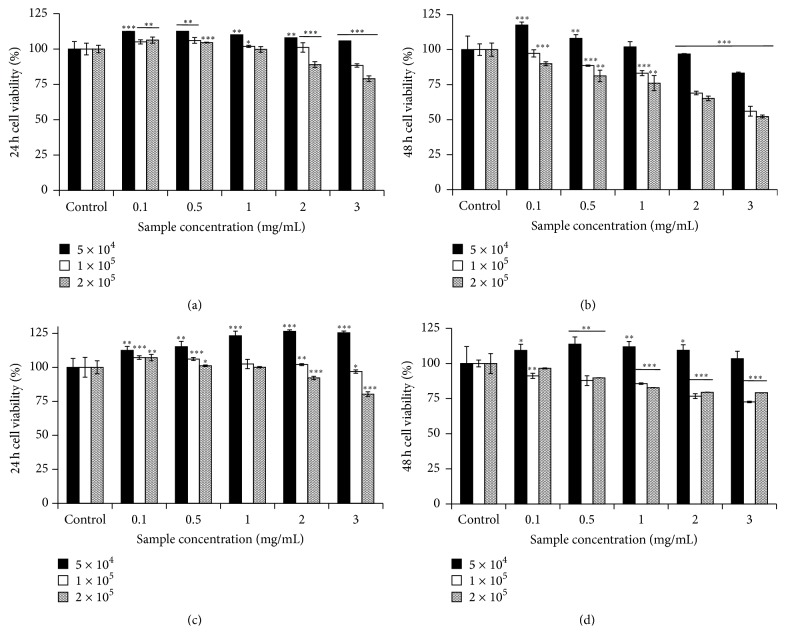
Cell viability of RAW 264.7 treated with PFSP extracts: SP (Steamed, Peeled) for (a) 24 h and (b) 48 h and SNP (Steamed, No Peeled) for (c) 24 h and (d) 48 h. Each value is expressed as mean ± SD (*n* = 3). *∗* indicates *P* < 0.05, *∗∗* indicates *P* < 0.01, and *∗∗∗* indicates *P* < 0.001 versus control.

Consistent with the assumption of different results, the growth effect of both SP and SNP extracts on RAW 264.7 cells was correlated to cell number, extracts concentration, and incubation time. The results showed that PFSP extracts did not have cytotoxic effect on RAW 264.7 murine macrophage cells; they even increased the cell viability (5 × 10^4^ cells/mL) within 24 h (Figures 1(a) and 1(c)). In a higher concentration of RAW 264.7 cells (1 × 10^5^ and 2 × 10^5^ cells/mL), high amount of PFSP extracts could reduce macrophage cells viability. However, the viabilities still remained above 50% after 48 h incubation (Figures 1(b) and 1(d)).

Besides, higher number of macrophage cells had lower viability after 24 and 48 h incubation. In a high number of macrophage cells (1 × 10^5^ and 2 × 10^5^ cells/mL), low concentration of PFSP extracts might provide a matrix for cell anchorage and migration so that cell proliferation is enhanced. However, high concentration of anthocyanins in PFSP extracts may cause a marked inhibition of cell proliferation [41]. Furthermore, longer incubation time caused higher inhibition of cell proliferation. These phenomena may correlate to the space for cell growth and the nutrition in medium.

### 3.2. Effect of PFSP Extracts on RAW 264.7 Cell Migration

Plant secondary metabolites have been demonstrated as important sources of potential agents that modify the various steps of wound repair. Various groups of natural products belonging to the terpenoids, flavonoids, and other polyphenols, alkaloids, and so forth have been identified with potent wound healing effect, such as cell migration, both* in vitro* and* in vivo*. Some have been shown to act at singular targets while others act at multiple targets through nonspecific action [42]. Phenolic compounds have been documented to possess potent antioxidant and free radical scavenging effect, which is believed to be one of the most important components of wound healing. For example, the wound healing effect of tannins may also be attributed to their anti-inflammatory activity due to their antioxidant action [43]. It is generally believed that addition of some antioxidants to the wound microenvironment or in food would support the repair process [44].

**Figure 2:**
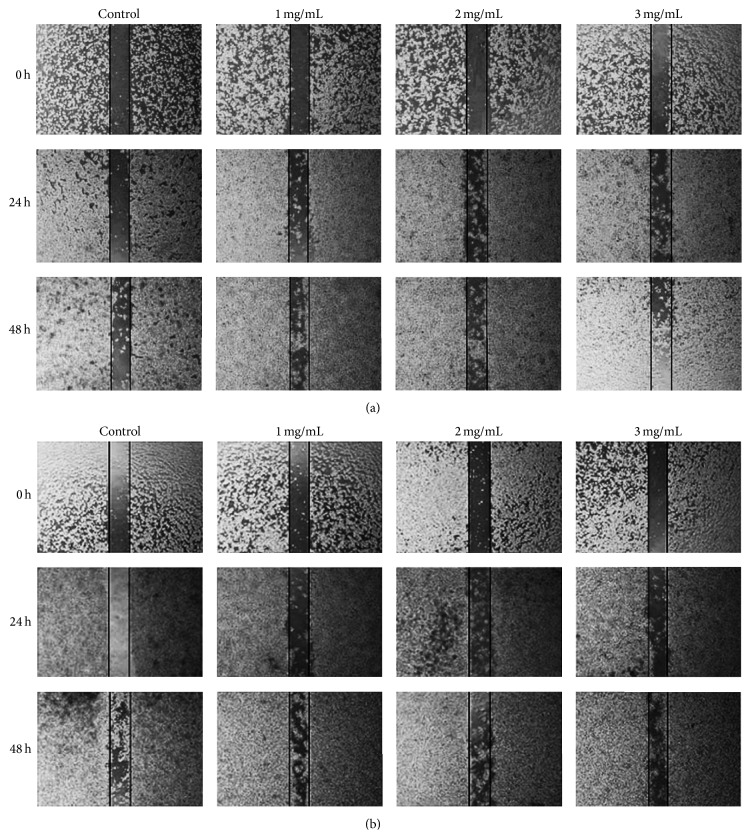
Effect of PFSP extracts: (a) SP (Steamed, Peeled) and (b) Steamed, No Peeled on RAW 264.7 cell migration after 24 h and 48 h incubation.

In accordance with previous studies, Figure 2 showed that PFSP extracts exhibited anti-inflammatory and promoted migration on LPS-induced macrophage cells in concentration- and time-dependent manner. However, there was no significant difference between the effect of SP and the effect of SNP extracts in increasing the macrophage cells migration. Macrophages produced high amount of nitric oxide (NO) in the presence of LPS, one of the most powerful inflammatory agents. NO is highly toxic for most pathogens but can also be highly damaging to neighboring cells. Thus, less macrophage cells could grow to fill the scratch. However, when PFSP extracts were added, M1 cell activation might diminish and immune response was converted to anti-inflammatory response so that more macrophage cells could migrate into the scratch.

### 3.3. Effect of PFSP Extracts on NO Production in LPS-Induced Macrophage Cell

In the past few years, NO has been a target of intensive research and drug development. NO is a gaseous signaling molecule that regulates various physiological and pathophysiological responses in the human body. To evaluate anti-inflammatory properties of PFSP extracts, RAW 264.7 murine macrophage cells were used because the cells can be activated by LPS to produce significant amount of nitric oxide (NO).

**Figure 3:**
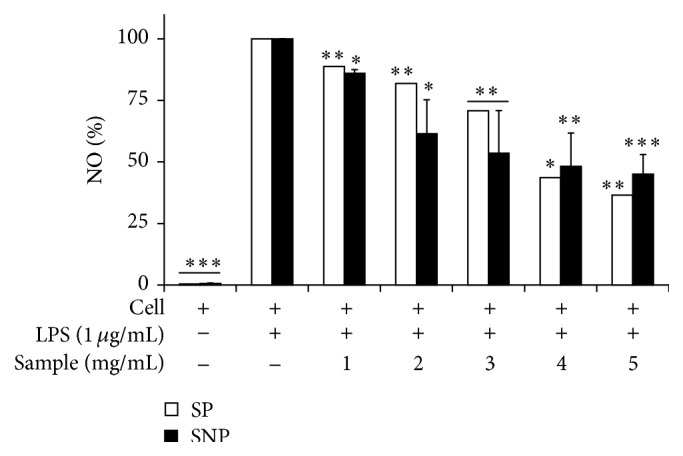
Inhibitory effect of PFSP extracts (SP (Steamed, Peeled) and SNP (Steamed, No Peeled)) on NO production in a culture medium of LPS-induced RAW 264.7 cells. Each value is expressed as mean ± SD (*n* = 3). *∗* indicates *P* < 0.05, *∗∗* indicates *P* < 0.01, and *∗∗∗* indicates *P* < 0.001 versus cells with LPS (negative control).

Both SP and SNP extracts under the dosage of no inhibitory effect on macrophage cell growth were capable of decreasing NO production by the LPS-activated RAW 264.7 cells (Figure 3). These results were in agreement with other studies describing anti-inflammatory activity of polyphenol, flavonoids, and anthocyanin from other plants. Anthocyanins contained in blackberry extract, containing cyanidin-3-O-glucoside, suppressed NO production through the downregulation of iNOS protein expression and the inhibition of LPS-induced NF-*κβ* activation [45]. Abundant levels of phenolic compounds including flavonoids, condensed tannins, and proanthocyanidins found in water and ethanolic extract of longan (*Dimocarpus longan* Lour.) flowers showed significant concentration-dependent inhibition of nitric oxide production. These inhibitory effects were further attributed to suppression of inducible nitric oxide synthase protein expression and not to reduced enzymatic activity [46]. Wang and Mazza [47] also reported that anthocyanins had strong inhibitory effects on NO production.

### 3.4. Inhibitory Effect of PFSP Extracts on the Expression of Proinflammatory Protein and Cytokines in LPS-Induced RAW 264.7 Cell

**Figure 4:**
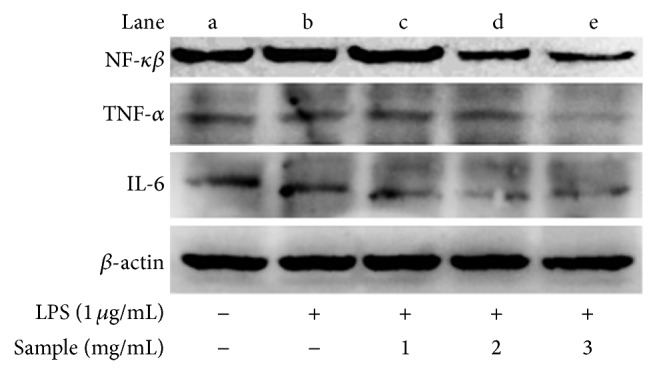
Inhibitory effect of SP (Steamed, Peeled) extracts on the expression of proinflammatory cytokines in LPS-induced RAW 264.7 cells.

Previous study from Hou et al. [48] has demonstrated that one of the flavonoid compounds (C3G aglycone-cyanidin) effectively inhibits iNOS and COX-2 expression in LPS-activated murine macrophages via blocking the activation of NF-*κβ*. Similarly, western blot results in this study revealed that SP extracts of PFSP TNG 73 could downregulate NF-*κβ* activation in LPS-induced RAW 264.7 cells (Figure 4). The treatment of RAW 264.7 cells with LPS alone (lane b) resulted in a minor increase in cytokine production relative to the control group (lane a). However, the protein levels of TNF-*α*, NF-*κβ*, and IL-6 in the supernatants from SP extracts-treated cells reduced significantly in a concentration-dependent manner relative to the LPS group (lane b). Furthermore, present study also demonstrated that SP extracts had inhibitory effects on the production of proinflammatory cytokines (TNF-*α* and IL-6). LPS-induced production of TNF-*α* and IL-6 was significantly inhibited by SP extracts in a concentration-dependent manner. These results suggested that SP extracts might have anti-inflammatory properties.

### 3.5. Effect of PFSP Extracts on Cancer Cell Viability

Pure anthocyanins and anthocyanin-rich extracts from fruits and vegetables have exhibited antiproliferative activity towards multiple cancer cell types* in vitro* [38, 39, 49–52]. Cell proliferation was inhibited by the ability of anthocyanins to block various stages of the cell cycle via effects on cell cycle regulator proteins (e.g., p53, p21, p27, cyclin D1, and cyclin A). Anthocyanidins appear to be more potent inhibitors of cell proliferation than the anthocyanins [52]. Interestingly, several investigations have compared the antiproliferative effects of anthocyanins on normal versus cancer cells and found that they selectively inhibit the growth of cancer cells with relatively little or no effect on the growth of normal cells.

As shown in Table 1, anthocyanins inhibited breast, colon, and stomach cancer cells proliferation in concentration- and time-dependent manner with IC_50_ (50% inhibitory concentration) value of approximately 3–7 mg/mL after 24 h treatment. These results were in accordance with previous studies that have reported about anticancer ability of anthocyanins. Olsson et al. [36] reported that 5 mg/mL of anthocyanin-rich extract from black currant could give 45% growth inhibition on MCF-7 cells. They also demonstrated that the same concentration of grape and bilberry extract containing anthocyanin also caused approximately 20–25% growth inhibition on breast cancer cells. Furthermore, Chen et al. [39] showed that cyanidin 3-glucoside purified from black rice (*Oryza sativa* L. indica) inhibited human breast cancer cell HS578T growth via G2/M arrest. Ding et al. [53] showed that cyanidin 3-glucoside could inhibit the proliferation, migration, and invasion of A549 lung tumor cells. Cyanidin [54] and cyanidin-3-rutinoside [55] were also found to increase the intracellular ROS level which may involve induction of apoptosis in human leukemic HL-60 cells. However, there is no previous publication of anthocyanins from purple sweet potato TNG 73 extracts regarding inhibiting the growth of MCF-7, SNU-1, and WiDr cancer cells.

### 3.6. Effect of PFSP Extracts on Cancer Cell Migration

**Figure 5:**
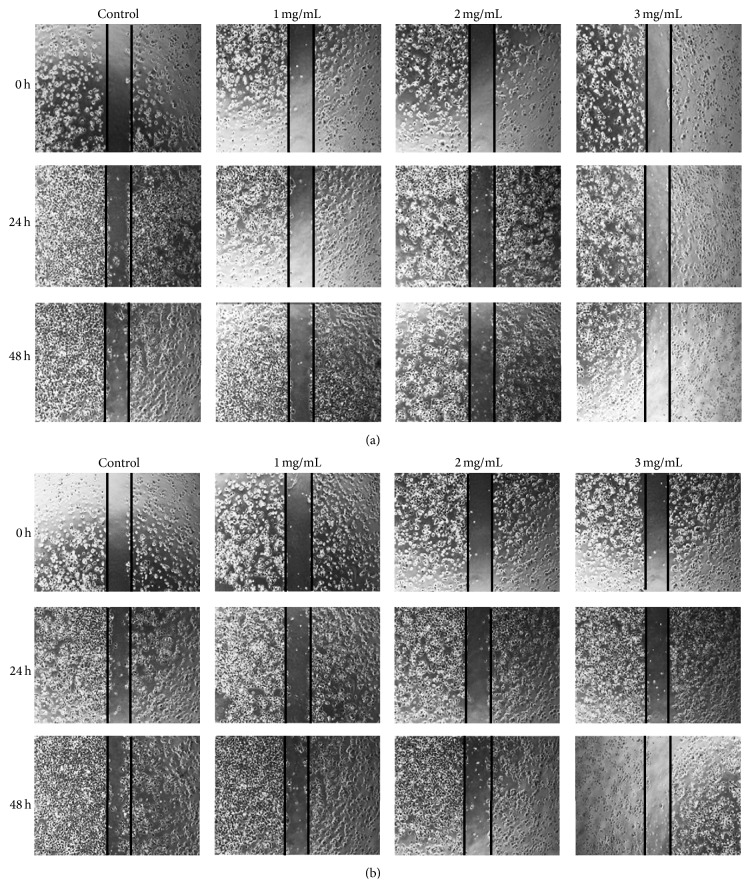
Effect of (a) SP (Steamed, Peeled) and (b) SNP (Steamed, No Peeled) extracts on MCF-7 cell migration after 24 h and 48 h incubation.

Motility is one of the properties in cancer cells that are needed for migration from the primary site to a secondary organ. Any alteration of this property would interrupt the metastatic cascade. Present study showed that PFSP TNG 73 extracts that contain high amount of antioxidative compounds, such as phenolics, flavonoids, and anthocyanins, effectively suppressed the migration of breast cancer cells (MCF-7) in a concentration-dependent manner (Figure 5). When confluent monolayers of cells were untreated with the extracts, MCF-7 cells could migrate to the gap after 24 h and more cells migrated after 48 h. Meanwhile, only a few of the treated cells could migrate into the gap. Previously, Lee et al. [56] reported that gingerol, phenolic substances found in ginger (*Zingiber officinale*), could reduce the motility of MDA-MB-231 human breast cancer cells. Another study by Li et al. [57] demonstrated that VI-12, a novel flavonoid derivative, could inhibit the migration and invasion of MDA-MB-231 and MDA-MB-435 human breast cancer cells.

### 3.7. Possible Apoptosis Pathways Caused by SP Extracts in Cancer Cell Lines MCF-7 and SNU-1

Apoptosis, or programmed cell death, plays a key role in the development and growth regulation of normal cells and is often dysregulated in cancer cells. Some of the most effective chemopreventive agents are strong inducers of apoptosis in premalignant and malignant cells. Anthocyanin-rich extracts from berries and grapes, and several pure anthocyanins and anthocyanidins, have exhibited proapoptotic effects in multiple cell types* in vitro* [36–40, 58]. The anthocyanins induce apoptosis through both intrinsic (mitochondrial) and extrinsic (FAS) pathways [40]. In addition, treatment of cancer cells, but not normal cells, with anthocyanins leads to an accumulation of ROS and subsequent apoptosis, suggesting that the ROS-mediated mitochondrial caspase-independent pathway is important for anthocyanin-induced apoptosis [55].

**Figure 6:**
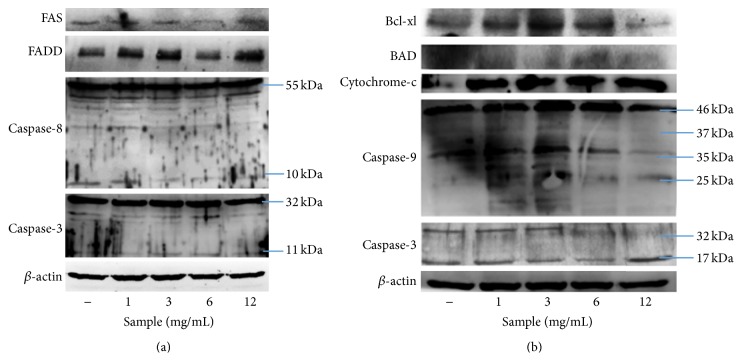
Expression of apoptotic proteins in SP extracts-treated MCF-7 cancer cell after 24 h incubation: (a) extrinsic and (b) intrinsic pathway.

**Figure 7:**
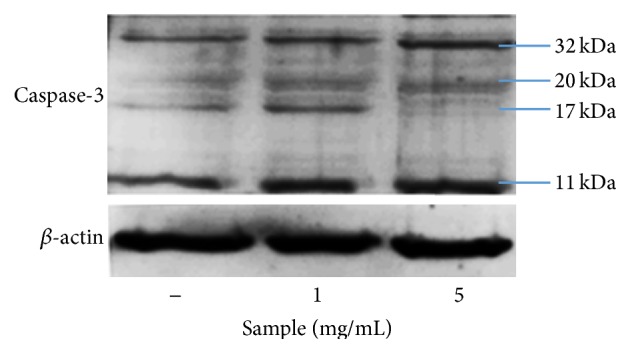
Expression of caspase-3 in SP extracts-treated SNU-1 cancer cell after 24 h incubation.

Correspondingly with previous studies, anthocyanins in SP extracts showed the ability to induce apoptosis on cancer cell line MCF-7 (Figure 6) through both the activation of FAS receptor (extrinsic pathway) and the release of cytochrome C (mitochondrial pathway). Meanwhile, the results in this study demonstrated that apoptosis in SNU-1 cancer cells was only induced through the activation of caspase-3 (Figure 7). Further study on the other pathways still needs to be done for this cancer cell line.

## 4. Conclusions

Purple-fleshed sweet potato (PFSP) Tainung 73 possesses high amount of antioxidative compounds, such as phenolics, flavonoid, and anthocyanin. The major anthocyanin is cyanidin or/and peonidin and their acylated derivatives. Study on the possible properties of PFSP extracts showed that these extracts had potential anti-inflammatory and anticancer activities. Anthocyanin-rich extracts of PFSP TNG 73 could suppress the production of nitric oxide (NO) and some proinflammatory cytokines, such as NF*κ*-*β*, TNF-*α*, and IL-6, in LPS-induced macrophage cell. Nevertheless, these extracts showed no cytotoxicity effect on macrophage cells. On the other hand, these extracts could inhibit the growth of some cancer cell lines, such as human breast cancer (MCF-7), gastric cancer (SNU-1), and colon adenocarcinoma (WiDr), in concentration- and time-dependent manner. After further investigation on molecular mechanism, PFSP TNG 73 extracts demonstrated the ability to induce apoptosis in MFC-7 cancer cell line through extrinsic and intrinsic pathways. Thus, PFSP TNG 73 can be used for future application of drugs, nutritional food, and health supplement.

## Figures and Tables

**Table 1:** IC_50_ of PFSP extracts (SP (Steamed, Peeled) and SNP (Steamed, No Peeled)) on MCF-7, WiDr, and SNU-1 cancer cell viability.

Cancer cell line	Sample concentration (mg/mL)					
	Steamed, Peeled (SP)			Steamed, No Peeled (SNP)		
	24 h	48 h	72 h	24 h	48 h	72 h
MCF-7	5.9	4.3	4.1	4.9	4.3	3.4
SNU-1	3.3	2.9	2.7	4.6	4.1	3.6
WiDr	6.9	6.1	5.9	7.1	6.6	4.6

MCF-7 = human breast cancer cell; SNU-1 = human gastric cancer cell; and WiDr = human colon carcinoma cell.
